# Burden of stroke in adolescents and young adults (aged 15–39 years) in South East Asia: a trend analysis from 1990 to 2021 based on the global burden of disease study 2021

**DOI:** 10.3389/fstro.2025.1503574

**Published:** 2025-02-21

**Authors:** Prakasini Satapathy, Shubham Chauhan, Shilpa Gaidhane, Ashok Kumar Bishoyi, G. Padma Priya, Karthikeyan Jayabalan, Swati Mishra, Shilpa Sharma, Ganesh Bushi, Muhammed Shabil, Rukshar Syed, Kamal Kundra, Navneet Dev, Sabah Ansar, Sanjit Sah, Quazi Syed Zahiruddin, Shailesh Kumar Samal, Diptismita Jena, Khang Wen Goh

**Affiliations:** ^1^Centre of Research Impact and Outcome, Chitkara University, Rajpura, Punjab, India; ^2^Faculty of Data Science and Information Technology, INTI International University, Nilai, Malaysia; ^3^Center for Global Health Research, Saveetha Medical College and Hospital, Saveetha Institute of Medical and Technical Sciences, Saveetha University, Chennai, India; ^4^One Health Center (COHERD), Jawaharlal Nehru Medical College, Datta Meghe Institute of Higher Education, Wardha, India; ^5^Marwadi University Research Center, Department of Microbiology, Faculty of Science Marwadi University, Rajkot, Gujarat, India; ^6^Department of Chemistry and Biochemistry, School of Sciences, JAIN (Deemed to be University), Bangalore, Karnataka, India; ^7^Department of Chemistry, Sathyabama Institute of Science and Technology, Chennai, Tamil Nadu, India; ^8^Department of Pharmacology, IMS and SUM Hospital, Siksha 'O' Anusandhan (Deemed to be University), Bhubaneswar, Odisha, India; ^9^Department of Pharmacy, Chandigarh Pharmacy College, Chandigarh Group of Colleges-Jhanjeri, Mohali, Punjab, India; ^10^School of Pharmaceutical Sciences, Lovely Professional University, Phagwara, India; ^11^University of Cyberjaya, Cyberjaya, Selangor Darul Ehsan, Malaysia; ^12^University Center for Research and Development, Chandigarh University, Mohali, Punjab, India; ^13^Medical Laboratories Techniques Department, AL-Mustaqbal University, Hillah, Babil, Iraq; ^14^IES Institute of Pharmacy, IES University, Bhopal, Madhya Pradesh, India; ^15^New Delhi Institute of Management, Tughlakabad Institutional Area, New Delhi, India; ^16^Department of Dermatology, Graphic Era Deemed to be University, Clement Town, Dehradun, India; ^17^Department of Clinical Laboratory Sciences, College of Applied Medical Sciences, King Saud University, Riyadh, Saudi Arabia; ^18^Department of Pediatrics, Dr. D. Y. Patil Medical College Hospital and Research Center, Dr. D. Y. Patil Vidyapeeth (Deemed-to-be-University), Pimpri, Pune, Maharashtra, India; ^19^Department of Public Health Dentistry, Dr. D. Y. Patil Medical College Hospital and Research Center, Dr. D. Y. Patil Vidyapeeth (Deemed-to-be-University), Pimpri, Pune, Maharashtra, India; ^20^Department of Medicine, Korea Universtiy, Seoul, Republic of Korea; ^21^South Asia Infant Feeding Research Network (SAIFRN), Division of Evidence Synthesis, Global Consortium of Public Health and Research, Datta Meghe Institute of Higher Education, Wardha, India; ^22^Unit of Immunology and Chronic Disease, Institute of Environmental Medicine, Karolinska Institute, Stockholm, Sweden; ^23^Noida Institute of Engineering and Technology (Pharmacy Institute), Greater Noida, India; ^24^Faculty of Mathematics and Natural Sciences, Universitas Negeri Padang, Padang, Indonesia

**Keywords:** stroke, ischemic stroke, intracerebral hemorrhage, subarachnoid hemorrhage, Southeast Asia, Global Burden of Disease, adolescents, young adults

## Abstract

**Background:**

Stroke is a leading cause of morbidity and mortality globally, yet its burden among adolescents and young adults (aged 15–39 years) in South East Asia (SEA) remains understudied. Understanding regional trends and risk factors in this population is critical for effective prevention and management strategies. This study aims to examine stroke trends from 1990 to 2021, focusing on ischemic stroke, intracerebral hemorrhage (ICH), and subarachnoid hemorrhage (SAH) in SEA.

**Methods:**

Using data from the Global Burden of Disease (GBD) 2021 study, temporal trends in stroke incidence, mortality, and Disability-Adjusted Life Years (DALYs) were analyzed for the age group 15–39 years. Join point regression analysis was employed to identify significant changes in stroke trends, and gender specific patterns were also assessed.

**Results:**

Ischemic stroke cases in SEA increased from 28030.85 to 40836.18, with a slight rise in incidence, particularly affecting males aged 30–39, while female mortality dropped by 23.81%. ICH incidence decreased annually by 0.6692%, with significant reductions in DALYs and mortality, especially among younger age groups and females. SAH incidence declined by 0.2142%, accompanied by a notable reduction in female mortality (31.83%). Countries with lower SDI experienced higher stroke incidence and mortality rates, highlighting socio-economic disparities. Geographic analysis revealed the Philippines had the highest rise in ischemic stroke, while most other countries saw declines in ICH and SAH rates.

**Conclusion:**

The study highlights significant progress in managing ICH and SAH, especially among younger populations and females. However, ischemic stroke remains a growing challenge, particularly for males, necessitating targeted interventions to reduce the overall stroke burden.

## Introduction

Stroke affects a significant portion of the global population, with up to one in five people experiencing it in high-income countries and nearly one in two in low-income countries (Hilkens et al., 2024). It is the second most common cause of death worldwide, with a marked rise in incidence in recent decades (Feigin et al., 2022). The burden of stroke is particularly severe in low and middle income countries (LMICs), where it accounts for 86% of global stroke-related deaths and 89% of DALYs lost (Feigin et al., 2022). While stroke is predominantly associated with older populations, recent global trends indicate a rising incidence among adolescents and young adults, aged 15–39 years (Brusca and Albert, 2023; Ma et al., 2024). This shift poses unique challenges, as stroke in younger populations can result in long-term disability, economic loss, and significant healthcare costs (Pandian et al., 2023).

SEA, a region characterized by rapid urbanization, changing lifestyle patterns, and evolving healthcare systems, has seen a dramatic rise in stroke cases among younger populations (Venketasubramanian et al., 2017; Potter et al., 2022; Bako et al., 2022; Béjot et al., 2016). Stroke is now the leading cause of death in many SEA countries, and its prevalence is projected to increase as risk factors such as hypertension, diabetes, smoking, and physical inactivity become more widespread in this region (Sebastian et al., 2023; Region AWitS-EA)^1^. Within SEA, disparities in healthcare access and quality further complicate the management of stroke (Apor et al., 2021; Kalkonde et al., 2023; Sebastian et al., 2023). Stroke can be classified into three major subtypes: ischemic stroke, ICH, and SAH, each of which presents distinct etiological and prognostic challenges (Feigin et al., 2021). Among these, ischemic stroke remains the most common subtype, particularly in younger populations, with growing evidence suggesting an increasing burden among males (Ma et al., 2024).

This study aims to provide a comprehensive analysis of stroke trends among adolescents and young adults (aged 15–39 years) in SEA from 1990 to 2021, using data from the GBD study. By examining the incidence, mortality, and DALYs associated with ischemic stroke, ICH, and SAH, this research offers valuable insights into the evolving stroke burden in this critical age group. The findings are expected to inform targeted public health strategies aimed at reducing the burden of stroke and improving healthcare outcomes in the region.

## Methods

### Data source and data collection

This study uses data from the Global Health Data Exchange (GHDx), sourced from the latest version of the GBD 2021 study (https://vizhub.healthdata.org/gbd-results/; Global Burden of Disease Collaborative Network, 2024). This analysis utilizes the Stroke data estimates provided by the Institute for Health Metrics and Evaluation (IHME). The GBD database is a globally comprehensive health database, that includes a diverse range of diseases and related health metrics (Feigin et al., 2024). This study focuses on the SEA region and data were collected for 13 SEA countries and territories such as Cambodia, Indonesia, Lao People's Democratic Republic, Malaysia, Maldives, Mauritius, Myanmar, Philippines, Seychelles, Sri Lanka, Thailand, Timor-Leste, Viet Nam. We selected overall stroke and its subtypes ischemic stroke, intracerebral hemorrhage, and subarachnoid hemorrhage in the age group of 15–39 years. For the analysis, we employed four standard epidemiological metrics: incidence, prevalence, mortality, and disability-adjusted life years (DALYs) from 1990 to 2021. Measures were stratified by sex, country, age, and SDI. QGIS (version 3.38) was used to describe the regional distribution of stroke in SEA. Researchers from the Global Burden of Disease (GBD) study have created the Sociodemographic Index (SDI), a composite measure that reflects a region's level of development and has a strong correlation with health outcomes. The SDI is calculated as the geometric mean and ranges from 0 to 1. Countries and regions are divided into five SDI (Sociodemographic Index) categories low (SDI < 0.45), low-middle (SDI 0.45–0.60), middle (SDI 0.61–0.68), high-middle (SDI 0.69–0.79), and high (SDI ≥ 0.80) to facilitate more accurate health-related estimations. More detailed methodology have been described elsewhere (Feigin et al., 2024, 2021). The Pearson correlation was employed to examine the relationship between ASIR, ASMR, and SDI, with statistical significance determined at *p* < 0.05.

### Case definitions

A comprehensive analysis was conducted on total strokes and specific subtypes: ischemic strokes (IS), intracerebral hemorrhage (ICH), and subarachnoid hemorrhage (SAH). Stroke, as defined by the WHO clinical criteria, refers to the rapid onset of clinical signs of neurological disturbance, which lasts for more than 24 h or causing death with vascular origin as the only apparent cause. IS were defined as a neurological dysfunction caused by focal infarction in the brain, spinal cord, or retina. Intracerebral hemorrhage is characterized by a focal accumulation of blood in the brain, not resulting from trauma. Subarachnoid hemorrhage is a type of non-traumatic stroke caused by bleeding into the subarachnoid space of the brain (Feigin et al., 2021). All subtypes of strokes were categorized using approved codes from the International Classification of Diseases, Ninth (ICD-9) and Tenth (ICD-10) editions. The Global Burden of Disease (GBD) study's estimates of stroke burden are restricted to first stroke event in a person's life. Detailed information on fatal and non-fatal estimates can be found at https://vizhub.healthdata.org/gbd-compare/ (Vos et al., 2020).

## Statistical analysis

### Join point regression analysis

In this study, Joinpoint regression analysis was employed to pinpoint significant trend shifts, allowing the identification of temporal changes in stroke burden. This analysis utilized the Joinpoint Regression Program (Version 5.2.0, National Cancer Institute), we systematically analyzed age-standardized incidence rates (ASIR), prevalence rates (ASPR), mortality rates (ASMR), and disability rates (ASDR) to locate statistically significant Joinpoints. The Annual Percent Change (APC) for each identified segment was computed, and the overall trend for the study period was encapsulated through the calculation of the Average Annual Percent Change (AAPC). The AAPC was computed using the following equation: AAPC = ∑ (Ti/T × APC_i_), where T_i_ is the duration of each segment, T represents the total study period, and APC_i_ indicates the annual percent change for each segment (Kumar et al., 2024). To ensure the statistical significance of observed changes and to mitigate the influence of random fluctuations, the Monte Carlo Permutation method was applied (Megeri et al., 2018; Kim et al., 2000; Swain et al., 2023). Additionally, we addressed potential heteroscedasticity where the variance of residuals varies across trend segments by examining variance patterns and applying appropriate transformations and modeling adjustments. One such adjustment involved the logarithmic transformation of the rates, formulated as log_(yt)__=_ β_0_ + β_1_t + ϵ_t_, where y_t_ are the measured rates, t represents time, and ϵ_t_ denotes the error terms. These steps ensured that our findings were statistically robust and provided an accurate depiction of the epidemiological transitions observed. All data analyses were performed using age-standardized rates and uncertainty intervals, further enhancing the reliability of the trends detected. Moreover, Pearson correlation analysis was conducted to assess the association between ASRs and SDI level.

### Ethical considerations

The data used in this study is publicly available and fully anonymized, sourced from the GHDx database. No new primary data collection involving human subjects was performed, and all analyses are consistent with ethical guidelines established in previous GBD research. This study ensured the responsible use of health data, with no direct involvement of individual patient information.

## Results

The analysis of stroke incidence in SEA from 1990 to 2021 reveals distinct trends across ischemic stroke, ICH, and SAH, with variations by sex and age groups.

### Ischemic stroke

Between 1990 and 2021, ischemic stroke cases in Southeast Asia (SEA) increased significantly. Cases rose from 28030.85 (95% CI: 19767.28 to 39164.77) to 40836.18 (95% CI: 29870.25 to 56962.88), with incidence rates increasing from 14.23 (95% CI: 10.03 to 19.88) to 14.72 (95% CI: 10.77 to 20.54) per 100,000 people. A broader dataset showed a similar rise in cases from 180705.69 (95% CI: 150771.71 to 211571.35) to 242,608.86 (95% CI: 197679.3 to 289164.37) (Tables 1, 2). Despite this, the DALYs rate showed a slight decline, with an AAPC of −0.1536% (95% CI: −0.1735% to −0.1334%). Gender-specific analysis indicated an increase in female cases from 16348.95 (95% CI: 11639.26 to 23026.88) to 22363.22 (95% CI: 16236.66 to 31306.66), while their DALYs rate decreased (APC: −13.95%, 95% CI: −25.35% to −2.44%). Male cases rose from 11681.91 (95% CI: 8077.41 to 16358.89) to 18472.96 (95% CI: 13524.97 to 25747.33), with an increase in their DALYs (APC: 8.34%, 95% CI: −9.63% to 30.41%). Mortality trends indicated rising female deaths, but their death rate declined (APC: −23.81%, 95% CI: −43.87% to −12.32%), while male death rates remained stable. Age-specific trends showed reductions in incidence, prevalence, and DALYs among younger age groups (15–39 years), with minimal improvements observed in older populations.

**Table 1:** The incidence and DALYs of stroke and theirs AAPCs from 1990 to 2021 in Southeast Asia.

	**Cases (*n*), 1990**	**Incidence rate (per 100,000 population), 1990**	**Cases (*n*), 2021**	**Incidence rate (per 100,000 population), 2021**	**APC 1990–2021**	**AAPC 1990–2021**	**Cases (*n*), 1990**	**DALYs rate (per 100,000 population), 1990**	**Cases (*n*), 2021**	**DALYs rate (per 100,000 population), 2021**	**APC 1990–2021**	**AAPC 1990–2021**
**Ischemic stroke**												
**Southeast Asia**	28030.85 (19767.28 to 39164.77)	14.23 (10.03 to 19.88)	40836.18 (29870.25 to 56962.88)	14.72 (10.77 to 20.54)	3.49 (10.54 to −3.57)	0.1078^*^ (0.0941 to 0.1172)	180705.69 (150771.71 to 211571.35)	91.73 (76.53 to 107.39)	242608.86 (197679.3 to 289164.37)	87.48 (71.28 to 104.27)	−4.63 (7.66 to −14.89)	−0.1536^*^ (−0.1735 to −0.1334)
**Sex**												
**Female**	16348.95 (11639.26 to 23026.88)	16.34 (11.63 to 23.01)	22363.22 (16236.66 to 31306.66)	16.39 (11.9 to 22.94)	0.31 (7.28 to −6.25)		104498.37 (82954.46 to 130805.55)	104.41 (82.88 to 130.69)	122623.77 (97910.44 to 151160.73)	89.84 (71.74 to 110.75)	−13.95 (−2.44 to −25.35)	−13.95 (−2.44 to −25.35)
**Male**	11681.91 (8077.41 to 16358.89)	12.05 (8.33 to 16.88)	18472.96 (13524.97 to 25747.33)	13.12 (9.6 to 18.28)	8.82 (18.73 to −0.2)		76207.33 (62565.9 to 90132.81)	78.63 (64.55 to 93)	119985.09 (92974 to 149027.58)	85.19 (66.01 to 105.81)	8.34 (30.41 to −9.63)	8.34 (30.41 to −9.63)
**Age group, Years**												
**15–19**	4061.67 (1854.64 to 7107.09)	8.26 (3.77 to 14.46)	4505.02 (2112.86 to 7802.8)	7.96 (3.73 to 13.78)	−3.71 (5.79 to −9.38)		20718.65 (16379.61 to 25413.59)	42.15 (33.32 to 51.7)	21047.41 (16607.57 to 25695.02)	37.17 (29.33 to 45.38)	−11.81 (2.07 to −24.07)	
**20–24**	4634.53 (2517.86 to 7494.89)	10.4 (5.65 to 16.82)	5669.44 (3180.54 to 8939.22)	10.07 (5.65 to 15.88)	−3.19 (3.67 to −7.63)		27461.11 (22240.05 to 33462.02)	61.62 (49.91 to 75.09)	29932.76 (23902.93 to 36576.68)	53.16 (42.45 to 64.96)	−13.74 (−1.41 to −25.95)	
**25–29**	5327.13 (2863.24 to 9009.77)	13.27 (7.13 to 22.44)	7136.96 (3972.96 to 11660.41)	12.56 (6.99 to 20.51)	−5.36 (0.1 to −10.41)		34924.52 (28396.97 to 42164.99)	86.97 (70.72 to 105.01)	42290.68 (34009.93 to 51302.1)	74.4 (59.83 to 90.25)	−14.46 (−0.41 to −25.52)	
**30–34**	6483.41 (4053.55 to 9867.19)	18.63 (11.65 to 28.36)	9882.02 (6434.28 to 14728.44)	17.89 (11.65 to 26.66)	−4 (2.03 to −9.92)		43200.82 (35543.79 to 51142.93)	124.17 (102.16 to 147)	60208.37 (48356.88 to 72259.44)	108.99 (87.54 to 130.81)	−12.22 (1.44 to −24.24)	
**35–39**	7524.12 (4192.3 to 12295.64)	26.55 (14.79 to 43.39)	13642.73 (7932.1 to 21381.37)	26.08 (15.16 to 40.87)	−1.77 (9.79 to −11.02)		54400.6 (45688.84 to 62871.21)	191.95 (161.21 to 221.84)	89129.64 (73055.5 to 106410.91)	170.37 (139.65 to 203.41)	−11.24 (3.24 to −22.67)	
**Southeast Asia**	32943.21 (25604.58 to 40700.35)	16.72 (13 to 20.66)	38204.61 (30528.44 to 45946.57)	13.78 (11.01 to 16.57)	−17.62 (−12.08 to −23.11)	−0.6692^*^ (−0.7226 to −0.6338)	940966.12 (847657.5 to 1032982.2)	477.64 (430.27 to 524.34)	1060304.71 (928870.27 to 1234604.5)	382.33 (334.94 to 445.18)	−19.95 (−5.2 to −31.98)	−0.7158^*^ (−0.7481 to −0.6762)
**Sex**												
**Female**	15325.04 (11554.07 to 19333.49)	15.31 (11.54 to 19.32)	14826.64 (11580.34 to 18165.65)	10.86 (8.48 to 13.31)	−29.05 (−24.49 to −33.75)		453775.81 (372802.43 to 547581.54)	453.39 (372.48 to 547.11)	385434.08 (325346.3 to 472787.13)	282.4 (238.38 to 346.41)	−37.71 (−19.45 to −51.53)	
**Male**	17618.17 (13834.33 to 21823.01)	18.18 (14.27 to 22.52)	23377.97 (19063.17 to 28095.19)	16.6 (13.54 to 19.95)	−8.69 (−2.74 to −14.68)		487190.31 (415700.73 to 553554.12)	502.68 (428.91 to 571.15)	674870.63 (567904.27 to 814339.7)	479.17 (403.22 to 578.19)	−4.68 (16.53 to −21.11)	
**Age group, Years**												
**15–19**	3077.43 (1792.27 to 4931.22)	6.26 (3.65 to 10.03)	2908.68 (1768.48 to 4494.67)	5.14 (3.12 to 7.94)	−17.94 (−7.96 to −25.15)		92247.12 (78190.54 to 108999.16)	187.67 (159.07 to 221.75)	80804.72 (69767.94 to 92686.76)	142.72 (123.23 to 163.71)	−23.95 (−5.26 to −39.07)	
**20–24**	4091.14 (2833.7 to 5832.38)	9.18 (6.36 to 13.09)	4063.06 (2847.84 to 5629.74)	7.22 (5.06 to 10)	−21.4 (−14.51 to −27.79)		118001.66 (100277.26 to 138907.53)	264.8 (225.02 to 311.71)	109818.03 (94359.89 to 128814.88)	195.03 (167.57 to 228.76)	−26.35 (−8.27 to −41.43)	
**25–29**	5425.02 (3574.56 to 7927.7)	13.51 (8.9 to 19.74)	5571.95 (3815.08 to 7784.87)	9.8 (6.71 to 13.7)	−27.45 (−20.27 to −34.68)		156203.19 (138585.96 to 176316.56)	389 (345.13 to 439.09)	152046.34 (130230.33 to 181418.13)	267.48 (229.1 to 319.15)	−31.24 (−16.42 to −43.17)	
**30–34**	8502.36 (6367.81 to 10914.11)	24.44 (18.3 to 31.37)	9707.83 (7537.91 to 12393.59)	17.57 (13.65 to 22.44)	−28.09 (−23.22 to −32.99)		231550.8 (206105.07 to 257298.89)	665.53 (592.39 to 739.54)	262944.57 (226147.56 to 312395.41)	476 (409.39 to 565.52)	−28.48 (−13.28 to −39.96)	
**35–39**	11847.26 (8299.1 to 15972.27)	41.8 (29.28 to 56.36)	15953.09 (11865.85 to 20929.95)	30.49 (22.68 to 40.01)	−27.05 (−19.95 to −32.58)		342963.36 (310909.62 to 380543.48)	1210.15 (1097.05 to 1342.75)	454691.06 (399404.15 to 528428.81)	869.15 (763.47 to 1010.1)	−28.18 (−13.66 to −38.66)	
**Southeast Asia**	32943.21 (25604.58 to 40700.35)	16.72 (13 to 20.66)	38204.61 (30528.44 to 45946.57)	13.78 (11.01 to 16.57)	−6.43 (−1.4 to −10.76)	−0.2142^*^ (−0.222 to −0.2063)	201564.52 (165935.5 to 241455.52)	102.31 (84.23 to 122.56)	224383.73 (185475.76 to 264836.92)	80.91 (66.88 to 95.5)	−20.92 (−4.37 to −33.4)	−0.7475^*^ (−0.7748 to −0.7092)
**Sex**												
**Female**	5790.62 (4314.09 to 7412.7)	5.79 (4.31 to 7.41)	7063.14 (5312 to 8917.32)	5.18 (3.89 to 6.53)	−10.55 (−5.83 to −14.43)		93044.35 (55465.11 to 111329.11)	92.96 (55.42 to 111.23)	89185.87 (72075.24 to 113273.88)	65.35 (52.81 to 82.99)	−29.71 (12.32 to −43.87)	
**Male**	17618.17 (13834.33 to 21823.01)	18.18 (14.27 to 22.52)	23377.97 (19063.17 to 28095.19)	16.6 (13.54 to 19.95)	−2.3 (3.75 to −7.8)		108520.17 (76268.87 to 147367.38)	111.97 (78.69 to 152.05)	135197.86 (99481.21 to 173118.22)	95.99 (70.63 to 122.92)	−14.27 (13.62 to −32.17)	
**Age group, Years**												
**15–19**	1191.2 (656.46 to 1895.17)	2.42 (1.34 to 3.86)	1253.54 (711.72 to 2035.01)	2.21 (1.26 to 3.59)	−8.64 (−1.18 to −14.91)		25714.72 (19722.07 to 31520.35)	52.32 (40.12 to 64.13)	22410.55 (17978.47 to 26957.76)	39.58 (31.75 to 47.61)	−24.34 (−3.5 to −39.15)	
**20–24**	1620.62 (1078.28 to 2297.28)	3.64 (2.42 to 5.16)	1825.11 (1234.17 to 2575.83)	3.24 (2.19 to 4.57)	−10.87 (−5.31 to −15.63)		31394.15 (25479.73 to 37600.24)	70.45 (57.18 to 84.37)	30111.49 (24813.37 to 35949.88)	53.47 (44.07 to 63.84)	−24.09 (−3.01 to −36.74)	
**25–29**	2193.3 (1370.68 to 3132.54)	5.46 (3.41 to 7.8)	2655.97 (1708.84 to 3662.36)	4.67 (3.01 to 6.44)	−14.46 (−7.95 to −19.45)		37254.8 (30692.9 to 45683.15)	92.78 (76.44 to 113.77)	38855.89 (31178.56 to 47789.36)	68.35 (54.85 to 84.07)	−26.32 (−6.81 to −40.18)	
**30–34**	2874.52 (2011.65 to 3864.27)	8.26 (5.78 to 11.11)	3839.19 (2750.92 to 5139.36)	6.95 (4.98 to 9.3)	−15.88 (−10.8 to −19.9)		45321.07 (36215.04 to 58497.65)	130.26 (104.09 to 168.14)	52566.88 (42137.91 to 64715.78)	95.16 (76.28 to 117.15)	−26.95 (−10.15 to −40.01)	
**35–39**	3406.77 (2119.42 to 4797.45)	12.02 (7.48 to 16.93)	5291.97 (3443.08 to 7249.85)	10.12 (6.58 to 13.86)	−15.85 (−6.79 to −22.03)		61879.78 (48923.58 to 78635.32)	218.34 (172.63 to 277.47)	80438.92 (64992.8 to 99289.71)	153.76 (124.24 to 189.79)	−29.58 (−11.35 to −43.34)	

^*^Indicates that AAPC is significantly different from zero at the alpha = 0.05 level.

**Table 2:** The prevalence and deaths of stroke and theirs AAPCs from 1990 to 2021 in Southeast Asia.

	**Cases (*n*), 1990**	**Prevalence (per 100,000 population), 1990**	**Cases (*n*), 2021**	**Prevalence (per 100,000 population), 2021**	**APC 1990–2021**	**AAPC, 1990–2021**	**Cases (*n*), 1990**	**Deaths (per 100,000 population), 1990**	**Cases (*n*), 2021**	**Deaths (per 100,000 population), 2021**	**APC 1990–2021**	**AAPC, 1990–2021**
**Ischemic stroke**												
Southeast Asia	439823.62 (382368.9 to 500015.39)	223.26 (194.09 to 253.81)	606079.86 (531395.8 to 677936.66)	218.54 (191.61 to 244.45)	−2.11 (0.54 to −4.88)	−0.0700^*^ (−0.0736 to −0.0668)	1655.64 (1368.12 to 2039.5)	0.84 (0.69 to 1.04)	2216.18 (1681.37 to 2768.83)	0.8 (0.61 to 1)	−4.63 (7.66 to −14.89)	−0.1284^*^ (−0.1688 to −0.088)
**Sex**												
Female	262733.2 (227080.16 to 300363.76)	262.51 (226.89 to 300.11)	348405.66 (305350.57 to 390986.77)	255.27 (223.73 to 286.47)	−2.76 (0.06 to −5.59)		312.86 (235.82 to 439.8)	2.19 (1.65 to 3.08)	400.15 (299.42 to 531.75)	1.54 (1.16 to 2.05)	−13.95 (−2.44 to −25.35)	
Male	177090.42 (154054.53 to 200587.08)	182.72 (158.95 to 206.96)	257674.2 (226226.52 to 288039.3)	182.95 (160.62 to 204.51)	0.13 (3.4 to −3.41)		314.83 (230.44 to 399.1)	2.24 (1.64 to 2.83)	592.84 (392.54 to 797.63)	2.25 (1.49 to 3.02)	8.34 (30.41 to −9.63)	
**Age group, Years**												
15–19	58591.76 (47833.71 to 70304.57)	119.2 (97.31 to 143.03)	58870.49 (49117.1 to 69088.3)	103.98 (86.75 to 122.03)	−12.77 (−10.12 to −15.61)		133.57 (101.25 to 175.18)	0.27 (0.21 to 0.36)	136.26 (104.1 to 174.02)	0.24 (0.18 to 0.31)	−11.43 (12.57 to −31.08)	
20–24	73581.85 (61548.52 to 86808.33)	165.12 (138.11 to 194.8)	83355.77 (71118.56 to 96240.84)	148.03 (126.3 to 170.91)	−10.35 (−7.66 to −12.97)		200.37 (152.63 to 266.61)	0.45 (0.34 to 0.6)	210.29 (160.73 to 265.6)	0.37 (0.29 to 0.47)	−16.94 (5.84 to −37.85)	
25–29	89516.11 (76267.78 to 103824.41)	222.93 (189.93 to 258.56)	115002.4 (99754.91 to 132022.24)	202.31 (175.49 to 232.25)	−9.25 (−6.63 to −11.71)		285.88 (231.43 to 366.16)	0.71 (0.58 to 0.91)	326.93 (246.94 to 411.71)	0.58 (0.43 to 0.72)	−19.21 (3.67 to −36.63)	
30–34	103459.43 (88836.75 to 120410.67)	297.37 (255.34 to 346.09)	151045.7 (132047.45 to 172834.03)	273.43 (239.04 to 312.88)	−8.05 (−5.41 to −10.4)		408.14 (340.93 to 503.5)	1.17 (0.98 to 1.45)	549.7 (411.21 to 698.25)	1 (0.74 to 1.26)	−15.17 (7.19 to −34.7)	
35–39	114674.47 (99167.82 to 130884.01)	404.63 (349.91 to 461.83)	197805.51 (173460.57 to 223900.39)	378.11 (331.57 to 427.99)	−6.55 (−4.05 to −8.88)		627.69 (514.83 to 759.38)	2.21 (1.82 to 2.68)	993 (755.63 to 1261.55)	1.9 (1.44 to 2.41)	−14.3 (7.75 to −32.34)	
Southeast Asia					−13.4 (−9.7 to −16.29)	−0.4778^*^ (−0.5013 to −0.4604)	15081.62 (13630.1 to 16734)	7.66 (6.92 to 8.49)	17191.06 (14871.77 to 20248.69)	6.2 (5.36 to 7.3)	−19.95 (−5.2 to −31.98)	−0.6701^*^ (−0.7074 to −0.6273)
**Sex**												
Female	7145.39 (5801.19 to 8654.72)	7.14 (5.8 to 8.65)	6054.66 (5033.81 to 7551.63)	4.44 (3.69 to 5.53)	−17.76 (−14.27 to −20.72)		7145.39 (5801.19 to 8654.72)	7.14 (5.8 to 8.65)	6054.66 (5033.81 to 7551.63)	4.44 (3.69 to 5.53)	−37.71 (−19.45 to −51.53)	
Male	7936.23 (6731.47 to 9081.58)	8.19 (6.95 to 9.37)	11136.39 (9330.36 to 13626.3)	7.91 (6.62 to 9.67)	−8.88 (−4.77 to −12.21)		7936.23 (6731.47 to 9081.58)	8.19 (6.95 to 9.37)	11136.39 (9330.36 to 13626.3)	7.91 (6.62 to 9.67)	−4.68 (16.53 to −21.11)	
**Age group, Years**												
15–19	38191.09 (31435.15 to 45329.22)	77.7 (63.95 to 92.22)	33571.66 (28213.07 to 39376.26)	59.3 (49.83 to 69.55)	−23.68 (−20.78 to −26.59)		1171.57 (977.26 to 1404.62)	2.38 (1.99 to 2.86)	1025.1 (878.8 to 1188.58)	1.81 (1.55 to 2.1)	−24.04 (−3.58 to −40.32)	
20–24	47133.29 (39615.09 to 54992.93)	105.77 (88.9 to 123.4)	47453.61 (40378.4 to 54785.04)	84.27 (71.71 to 97.29)	−20.32 (−17.04 to −23.37)		1612.15 (1356.05 to 1934.68)	3.62 (3.04 to 4.34)	1489.39 (1263.46 to 1769.17)	2.64 (2.24 to 3.14)	−26.89 (−7.26 to −43.22)	
25–29	57913.14 (49581.39 to 67943.55)	144.22 (123.47 to 169.2)	66494.72 (57984.84 to 76692.33)	116.98 (102.01 to 134.92)	−18.89 (−15.61 to −21.85)		2315.84 (2050.92 to 2614.03)	5.77 (5.11 to 6.51)	2226.42 (1883.83 to 2672.01)	3.92 (3.31 to 4.7)	−32.09 (−15.99 to −44.3)	
30–34	69851.93 (60164.74 to 81175.91)	200.77 (172.93 to 233.32)	89939.94 (78202.52 to 103474.79)	162.82 (141.57 to 187.32)	−18.9 (−15.5 to −21.69)		3780.69 (3337.89 to 4229.45)	10.87 (9.59 to 12.16)	4264.06 (3620.2 to 5135.84)	7.72 (6.55 to 9.3)	−28.96 (−12.78 to −41.03)	
35–39	84187.79 (73031.87 to 97988.36)	297.06 (257.69 to 345.75)	124957.36 (109917.47 to 142532.05)	238.86 (210.11 to 272.45)	−19.59 (−16.24 to −22.2)		6201.37 (5595.12 to 6921.77)	21.88 (19.74 to 24.42)	8186.1 (7108.43 to 9587.6)	15.65 (13.59 to 18.33)	−28.49 (−13.33 to −39.46)	
Southeast Asia	119368.11 (104534.65 to 136958.06)	60.59 (53.06 to 69.52)	158296.87 (139444.89 to 178930.57)	57.08 (50.28 to 64.52)	−5.8 (−3.25 to −8.38)	−0.1945^*^ (−0.1985 to −0.1908)	2985.78 (2387.63 to 3652.11)	1.52 (1.21 to 1.85)	3293.65 (2631.5 to 4041.98)	1.19 (0.95 to 1.46)	−20.92 (−4.37 to −33.4)	−0.7661^*^ (−0.8007 to −0.7172)
**Sex**												
Female	64211.86 (56082.12 to 74284.54)	64.16 (56.03 to 74.22)	80572.49 (70983.57 to 91103.13)	59.03 (52.01 to 66.75)	−7.98 (−5.38 to −10.78)		1326.69 (686.32 to 1633.89)	1.33 (0.69 to 1.63)	1233.32 (964.46 to 1648.06)	0.9 (0.71 to 1.21)	−29.71 (12.32 to −43.87)	
Male	55156.25 (48158.61 to 63076.02)	56.91 (49.69 to 65.08)	77724.38 (68280.76 to 87621.07)	55.19 (48.48 to 62.21)	−3.03 (0.19 to −5.81)		1659.09 (1107.19 to 2325.5)	1.71 (1.14 to 2.4)	2060.32 (1489.41 to 2699.37)	1.46 (1.06 to 1.92)	−14.27 (13.62 to −32.17)	
**Age group, Years**												
15–19	14328.25 (11519.06 to 17869.16)	29.15 (23.43 to 36.35)	14335.18 (11754.01 to 17634.46)	25.32 (20.76 to 31.15)	−13.14 (−10.85 to −15.41)		316.08 (230.25 to 398.58)	0.64 (0.47 to 0.81)	270.76 (210.54 to 333.54)	0.48 (0.37 to 0.59)	−25.63 (−2.14 to −41.83)	
20–24	18143.93 (14946.34 to 22174.25)	40.71 (33.54 to 49.76)	20083.44 (16747.82 to 23960.33)	35.67 (29.74 to 42.55)	−12.4 (−10 to −15.31)		411.2 (319.56 to 505.15)	0.92 (0.72 to 1.13)	386.96 (310.71 to 468.94)	0.69 (0.55 to 0.83)	−25.53 (−1.22 to −39.54)	
25–29	23551.36 (20137.89 to 27782.08)	58.65 (50.15 to 69.19)	29164.1 (25105.23 to 34090.65)	51.31 (44.16 to 59.97)	−12.52 (−10.33 to −15.1)		519.9 (414.52 to 649.75)	1.29 (1.03 to 1.62)	528.7 (408.11 to 670.86)	0.93 (0.72 to 1.18)	−28.16 (−5.28 to −43.28)	
30–34	29389.46 (25513.46 to 33958.06)	84.47 (73.33 to 97.6)	40523.61 (35385.85 to 46535.55)	73.36 (64.06 to 84.24)	−13.16 (−10.98 to −15.32)		685.9 (527.74 to 909.88)	1.97 (1.52 to 2.62)	774.34 (602.58 to 983.25)	1.4 (1.09 to 1.78)	−28.9 (−9.17 to −43.33)	
35–39	33955.1 (29976.81 to 38317.14)	119.81 (105.77 to 135.2)	54190.53 (48295.16 to 61031.93)	103.59 (92.32 to 116.66)	−13.54 (−11.54 to −15.35)		1052.7 (814.59 to 1374.64)	3.71 (2.87 to 4.85)	1332.89 (1033.67 to 1707.88)	2.55 (1.98 to 3.26)	−31.41 (−10.78 to −46.09)	

^*^Indicates that AAPC is significantly different from zero at the alpha = 0.05 level.

### Intracerebral hemorrhage

Between 1990 and 2021, intracerebral hemorrhage (ICH) cases in Southeast Asia (SEA) increased from 32943.21 (95% CI: 25604.58 to 40700.35) to 38204.61 (95% CI: 30528.44 to 45946.57). The incidence rate declined from 16.72 (95% CI: 13.00 to 20.66) to 13.78 (95% CI: 11.01 to 16.57) per 100,000, with an AAPC of −0.6692% (95% CI: −0.7226% to −0.6338%). Among females, the incidence rate showed a significant reduction (APC: −29.05%, 95% CI: −33.75% to −24.49%), while males experienced a smaller decline (APC: −8.69%, 95% CI: −14.68% to −2.74%) (Tables 1, 2). The DALYs rate decreased from 477.64 (95% CI: 430.27 to 524.34) to 382.33 (95% CI: 334.94 to 445.18) per 100,000, with an AAPC of −0.7158% (95% CI: −0.7481% to −0.6762%). The largest reductions in DALYs were observed in females (APC: −37.71%, 95% CI: −51.53% to −19.45%), while males experienced a smaller decline (APC: −4.68%, 95% CI: −21.11% to 16.53%). Age-specific analysis showed significant reductions in younger populations (15–39 years), with DALYs decreasing by −23.95% to −31.24%. Prevalence rates decreased from 150.9 (95% CI: 136.30 to 167.34) to 130.68 (95% CI: 114.87 to 147.25) per 100,000, with an AAPC of −0.4778% (95% CI: −0.5013% to −0.4604%). Females experienced a more pronounced reduction (APC: −17.76%, 95% CI: −20.72% to −14.27%), compared to males (APC: −8.88%, 95% CI: −12.21% to −4.77%). The greatest reduction in prevalence was observed in the 15–19 age group (APC: −23.68%, 95% CI: −26.59% to −20.78%), with similar declines across the 20–39 age groups. Mortality rates fell from 7.66 (95% CI: 6.92 to 8.49) to 6.2 (95% CI: 5.36 to 7.30) per 100,000, with an AAPC of −0.6701% (95% CI: −0.7074% to −0.6273%). Females showed a substantial reduction in mortality rates (APC: −37.86%, 95% CI: −51.53% to −19.45%), while males had a smaller decline (APC: −3.44%, 95% CI: −12.21% to 4.77%). The 15–19 age group showed the largest decline in mortality rates (APC: −43.7%, 95% CI: −39.15% to −5.26%), followed by significant declines in the 20–39 age groups (APC: −43.14% to −49.32%).

### Subarachnoid hemorrhage

Between 1990 and 2021, subarachnoid hemorrhage cases in Southeast Asia (SEA) increased, but key health metrics, including incidence, DALYs, prevalence, and mortality rates, consistently declined. The incidence rate decreased from 16.72 (95% CI: 13.00 to 20.66) to 13.78 (95% CI: 11.01 to 16.57) per 100,000, with an AAPC of −0.2142% (95% CI: −0.222% to −0.2063%). A sharper decline was observed in females (APC: −10.55%, 95% CI: −14.43% to −5.83%) compared to males (APC: −2.3%, 95% CI: −7.8% to 3.75%) (Tables 1, 2). Age-specific analysis showed reductions across all age groups, with declines ranging from −8.64% (95% CI: −14.91% to −1.18%) in younger populations to −15% (95% CI: −19.9% to −10.8%) in older groups. The DALYs rate declined significantly from 102.31 (95% CI: 84.23 to 122.56) to 80.91 (95% CI: 66.88 to 95.50) per 100,000, with an AAPC of −0.7475% (95% CI: −0.7748% to −0.7092%). Females experienced a larger reduction (APC: −29.71%, 95% CI: −43.87% to −12.32%) than males (APC: −14.27%, 95% CI: −32.17% to 13.62%). Among age groups, the steepest decline in DALYs was observed in the 30–39 age group (APC: −29.58%, 95% CI: −43.34% to −11.35%). Prevalence rates also showed a slight decrease from 60.59 (95% CI: 53.06 to 69.52) to 57.08 (95% CI: 50.28 to 64.52) per 100,000, with an AAPC of −0.1945% (95% CI: −0.1985% to −0.1908%). Females experienced a larger reduction (APC: −7.98%, 95% CI: −10.78% to −5.38%) compared to males (APC: −3.03%, 95% CI: −5.81% to 0.19%). Younger populations (15–39 years) experienced notable declines in prevalence rates. Mortality rates decreased, with the death rate falling from 1.52 (95% CI: 1.21 to 1.85) to 1.19 (95% CI: 0.95 to 1.46) per 100,000, with an AAPC of −0.7661% (95% CI: −0.8007% to −0.7172%). Females experienced a significant decline (APC: −31.83%, 95% CI: −43.87% to −12.32%), while males showed a smaller reduction (APC: −14.54%, 95% CI: −32.17% to 13.62%). The 15–19 age group recorded a decrease in mortality rates from 0.64 to 0.48 per 100,000 (APC: −12.7%, 95% CI: −15.41% to −10.85%).

**Figure 1:**
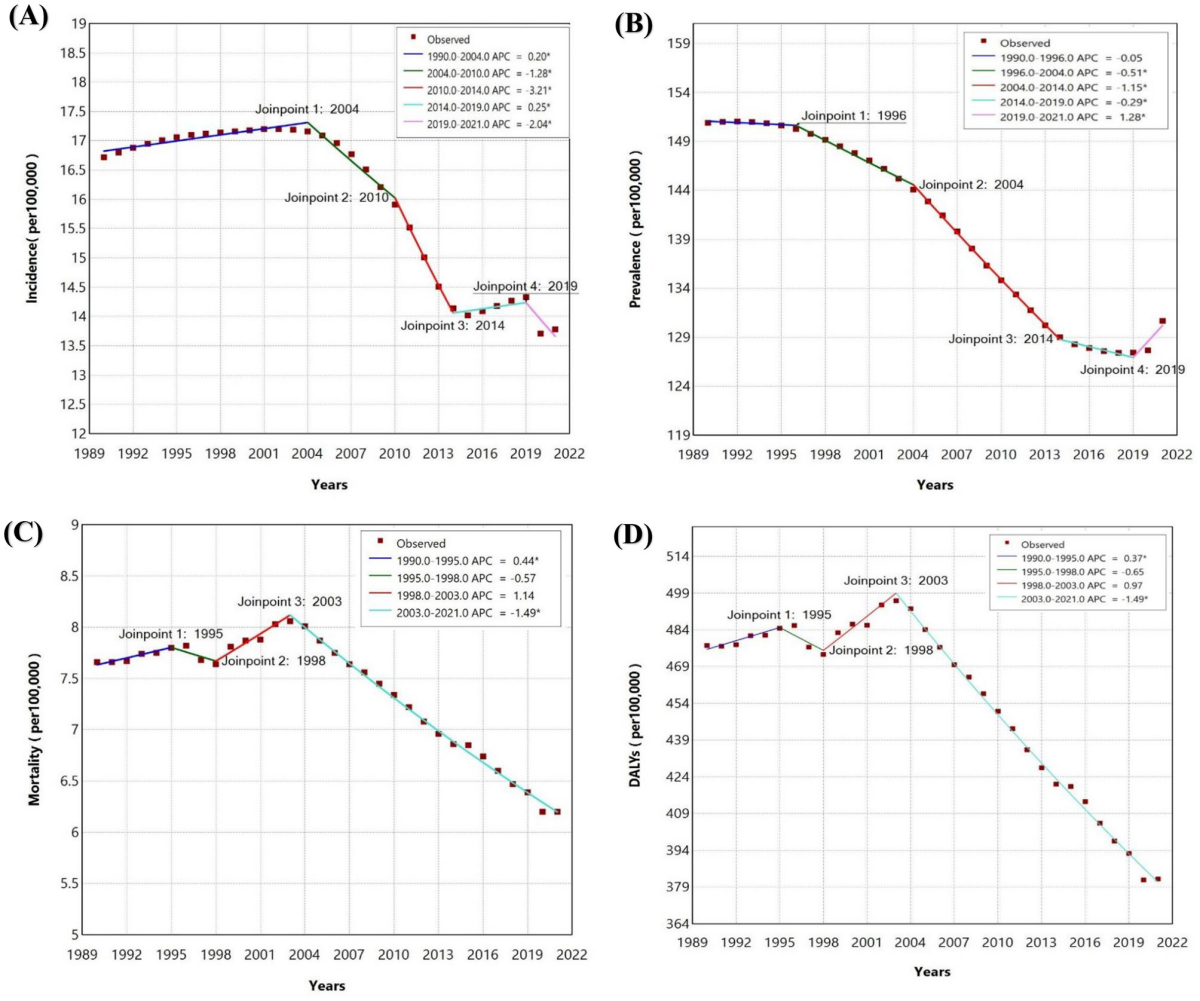
Join point regression analysis for Southeast Asia **(A)** Incidence Rate of Intracerebral haemorrhage **(B)** Prevalence Rate of Intracerebral haemorrhage **(C)** Mortality Rate of Intracerebral haemorrhage **(D)** DALYs Rate of Intracerebral haemorrhage in adolescents and young adults aged 15–39 years from 1990 to 2021.

Between 1990 and 2021, trends in intracerebral hemorrhage (ICH) among adolescents and young adults in Southeast Asia (SEA) showed fluctuations in incidence, prevalence, mortality, and DALYs rates. The incidence rate initially increased (APC: 0.20%, 95% CI: 0.12% to 0.29%) from 1990 to 2004, followed by significant declines between 2004 and 2010 (APC: −1.28%, 95% CI: −1.59% to −0.95%) and 2010 to 2014 (APC: −3.21%, 95% CI: −3.80% to −2.77%) (Figure 1). A small rise occurred between 2014 and 2019 (APC: 0.25%, 95% CI: 0.00% to 0.81%), followed by another significant decline from 2019 to 2021 (APC: −2.04%, 95% CI: −2.95% to −1.04%). Across the entire period, the AAPC for incidence was −0.67% (95% CI: −0.72% to −0.63%). Prevalence rates decreased slightly between 1990 and 1996 (APC: −0.05%, 95% CI: −0.14% to 0.09%), followed by intensified declines from 1996 to 2004 (APC: −0.51%, 95% CI: −0.60% to −0.44%) and 2004 to 2014 (APC: −1.15%, 95% CI: −1.22% to −1.10%). A moderate decline occurred between 2014 and 2019 (APC: −0.29%, 95% CI: −0.47% to −0.07%), with a slight increase in 2019–2021 (APC: 1.28%, 95% CI: 0.83% to 1.57%). The AAPC for prevalence across the entire period was −0.48% (95% CI: −0.50% to −0.46%). Mortality rates initially rose from 1990 to 1995 (APC: 0.44%, 95% CI: 0.07% to 1.10%) before showing consistent declines after 2003 (APC: −1.49%, 95% CI: −1.56% to −1.42%). Across the full period, the AAPC for mortality was −0.67% (95% CI: −0.71% to −0.63%). The DALYs rate showed early stability, with a small increase from 1990 to 1995 (APC: 0.37%, 95% CI: 0.02% to 0.95%), followed by significant declines from 2003 to 2021 (APC: −1.49%, 95% CI: −1.55% to −1.43%). The AAPC for DALYs over the entire period was −0.72% (95% CI: −0.75% to −0.68%).

**Figure 2:**
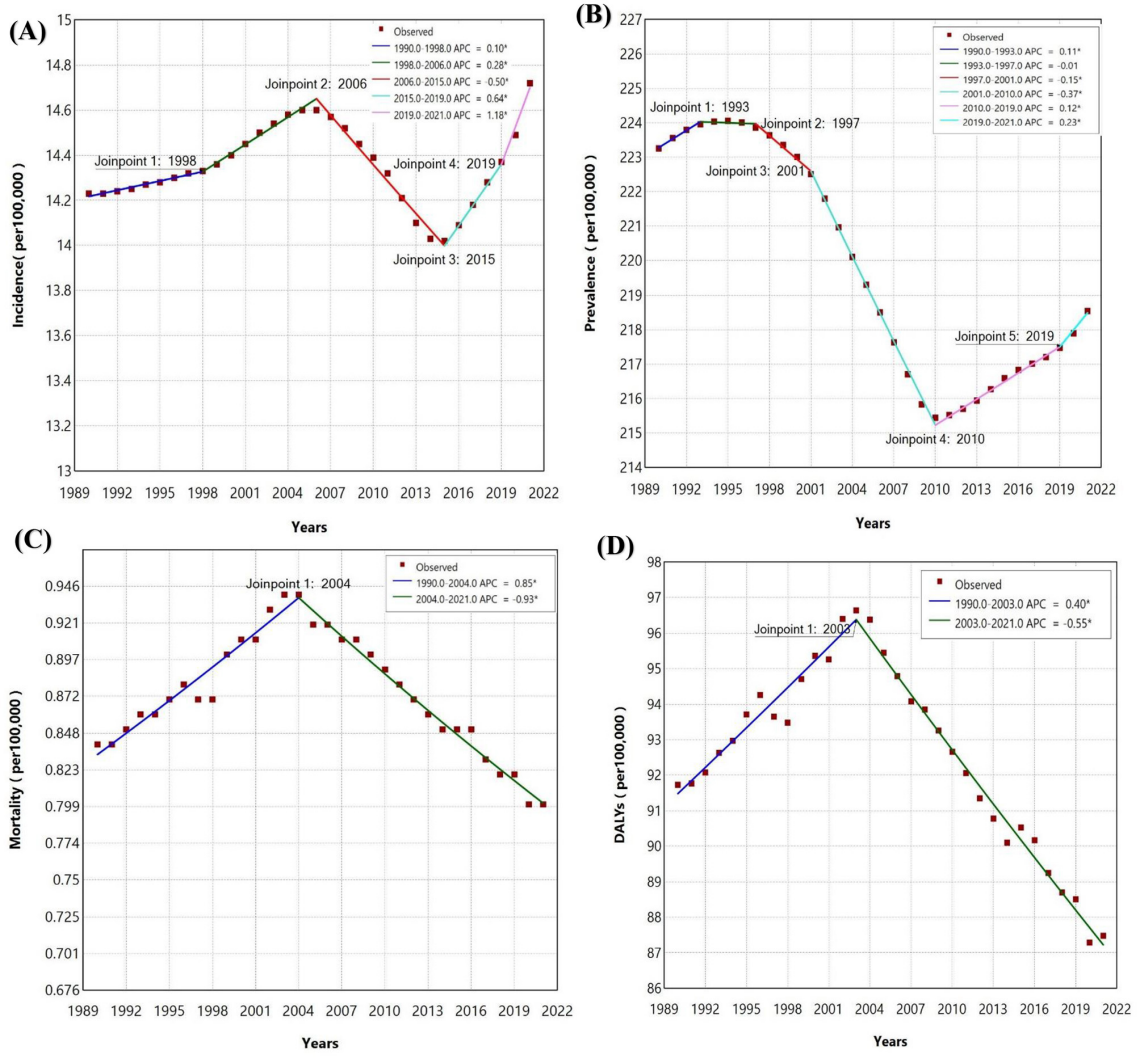
Join point regression analysis for Southeast Asia **(A)** Incidence of Ischemic stroke **(B)** Prevalence of Ischemic stroke **(C)** Mortality of Ischemic stroke **(D)** DALYs of Ischemic stroke in adolescents and young adults aged 15–39 years from 1990 to 2021.

The analysis of ischemic stroke reveals fluctuating trends across several metrics. Incidence initially rose modestly from 1990 to 1998 (APC = 0.10), increased more significantly until 2006 (APC = 0.28), then declined from 2006 to 2015 (APC = −0.50), before rising sharply through 2021 (Figure 2). Prevalence remained stable early on, with a minor decrease in 1997 (APC = −0.01), followed by a significant decline until 2010 (APC = −0.37). Prevalence rates then stabilized and slightly increased until 2019, with a sharper rise through 2021. Mortality rates rose until 2004 (APC = 0.85) but then declined continuously (APC = −0.93). DALYs followed a similar trend, increasing until 2003 (APC = 0.40) before significantly declining (APC = −0.55).

**Figure 3:**
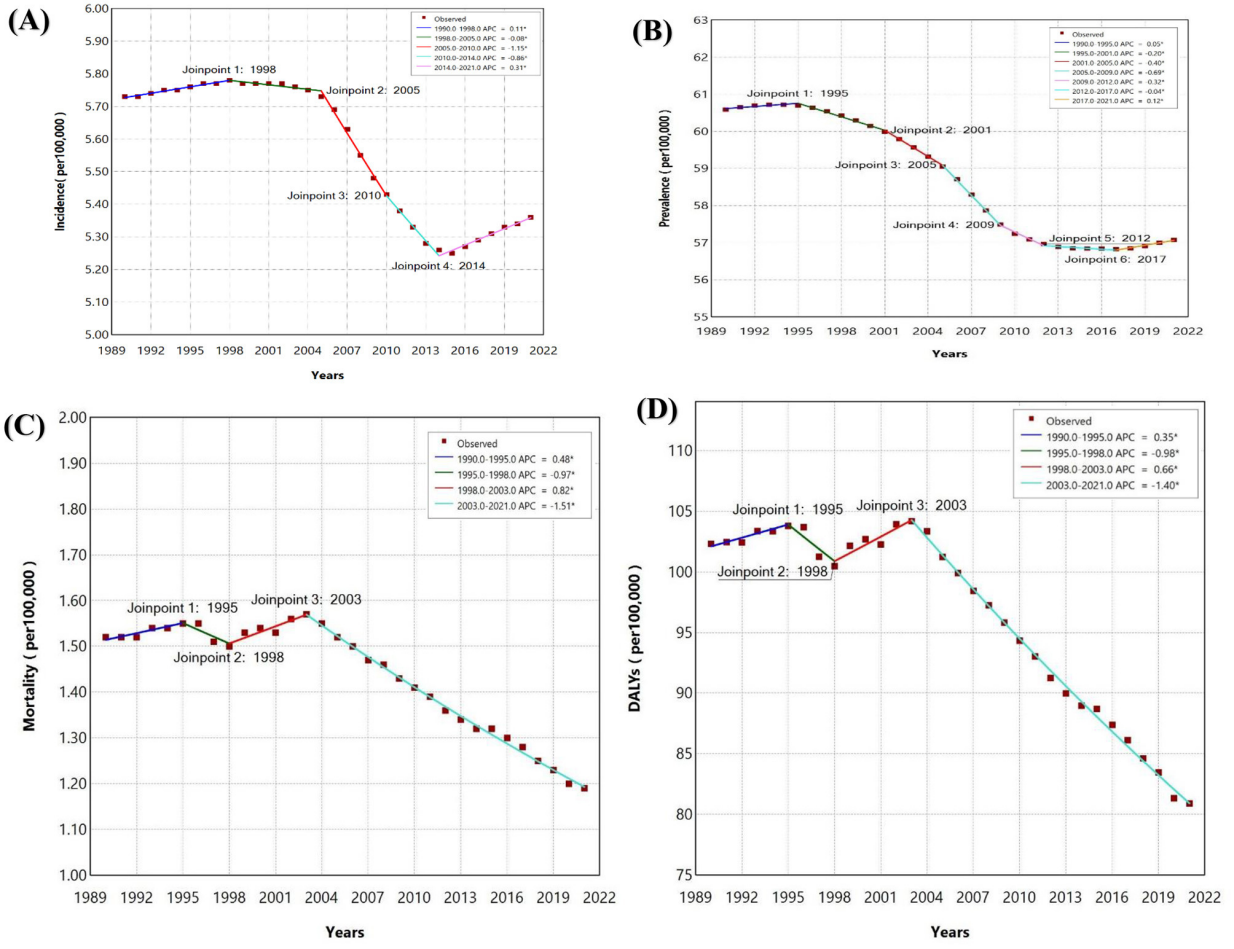
Join point regression analysis for Southeast Asia **(A)** Incidence of Subarachnoid hemorrhage **(B)** Prevalence of Subarachnoid hemorrhage **(C)** Mortality of Subarachnoid hemorrhage **(D)** DALYs of Subarachnoid hemorrhage in adolescents and young adults aged 15–39 years from 1990 to 2021.

The join point regression analysis for SAH from 1990 to 2021 shows significant trends across incidence, prevalence, mortality, and DALYs. SAH incidence initially increased (APC = 0.11% from 1990 to 1998) but declined significantly from 1998 to 2010, with the largest drop between 2005 and 2010 (APC = −1.15%) (Figure 3). The decline slowed until 2014 (APC = −0.86%) and then slightly increased from 2014 to 2021 (APC = 0.31%). Prevalence declined steadily after 1995, with the sharpest reduction from 2005 to 2009 (APC = −0.69%), followed by a small increase from 2017 to 2021 (APC = 0.12%). Mortality rose slightly from 1990 to 1995 (APC = 0.48%) but consistently declined after 2003 (APC = −1.51%). DALYs mirrored mortality trends, with a small increase from 1990 to 1995 (APC = 0.35%) and a sharp decline after 2003 (APC = −1.40%).

**Figure 4:**
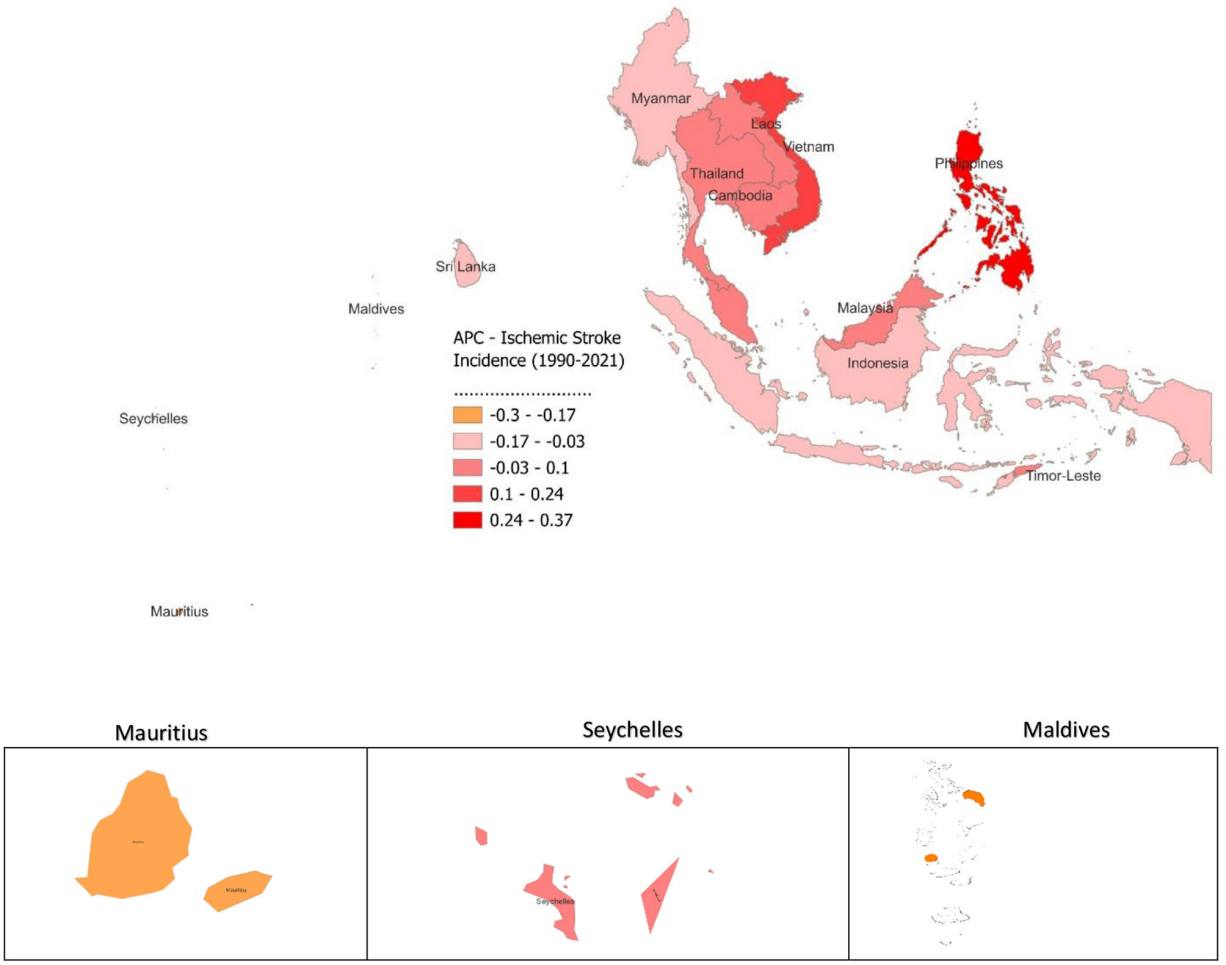
Annual Percentage Change (APC) of Ischemic Stroke Incidence from 1990 to 2021.

The analysis of APC in ischemic stroke incidence from 1990 to 2021 across SEA and smaller island nations reveals varying trends. The Philippines saw the highest increase, with an APC of 0.372, indicating a significant rise in cases. Other countries with rising trends include Vietnam (0.205), Thailand (0.084), Cambodia (0.046), Malaysia (0.029), Lao (0.019), and Timor-Leste (0.01), though these increases are smaller compared to the Philippines (Figure 4). In contrast, several countries showed declining trends, with Mauritius (−0.305) and Maldives (−0.203) reporting the largest decreases. Myanmar (−0.045), Sri Lanka (−0.051), Indonesia (−0.07), and Seychelles (−0.022) also saw modest reductions. The overall trend for SEA showed a slight positive APC of 0.035.

**Figure 5:**
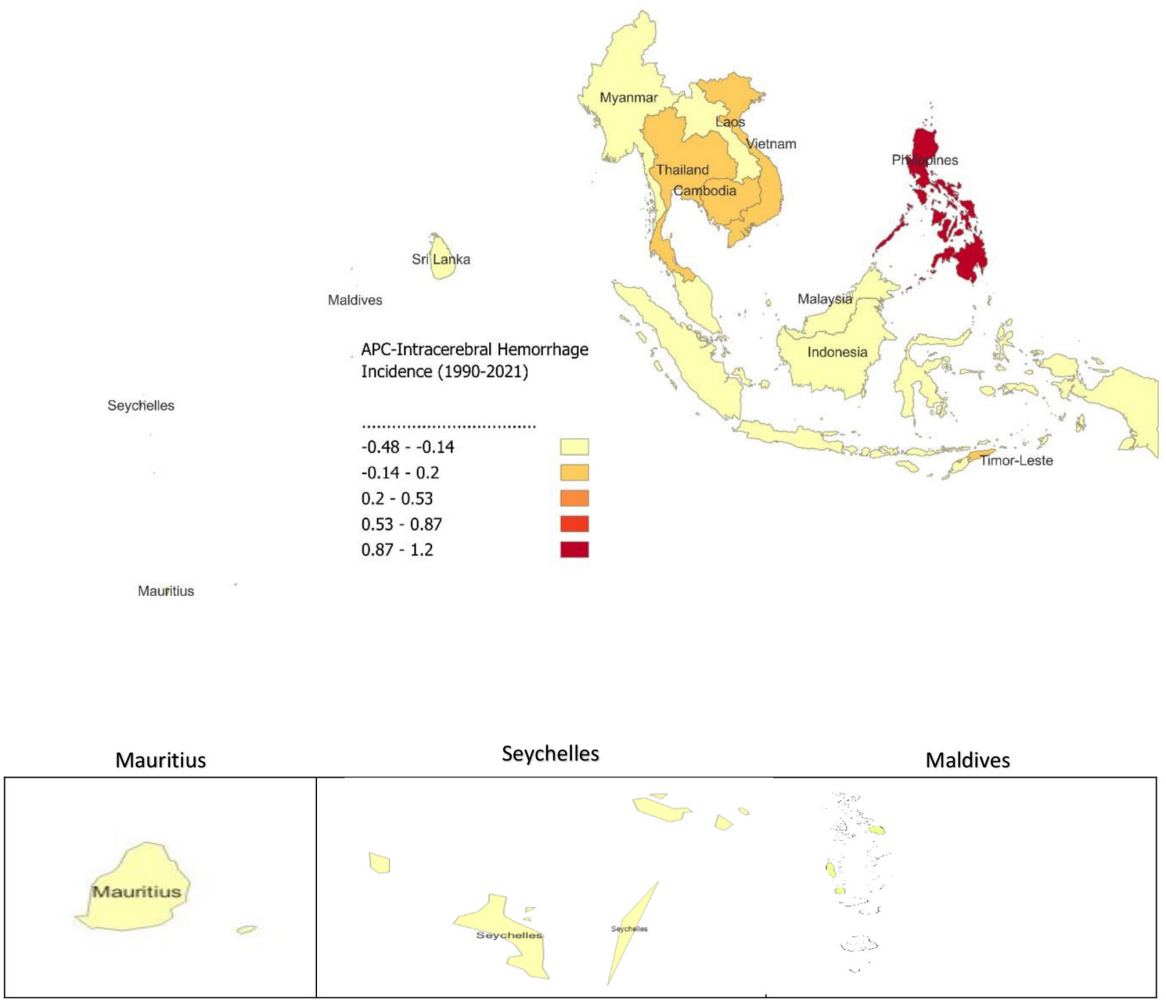
Annual Percentage Change (APC) of Intracerebral Hemorrhage Incidence from 1990 to 2021.

The analysis of APC in ICH incidence from 1990 to 2021 across SEA reveals varying trends. The Philippines shows the highest increase in ICH incidence, with an APC of 1.203 (Figure 5). In contrast, most other countries in the region report negative APC values, reflecting declines in ICH incidence. Notable decreases are seen in the Maldives (−0.477), Mauritius (−0.387), Sri Lanka (−0.37), Indonesia (−0.336), and Malaysia (−0.303). Countries like Lao PDR (−0.163), Myanmar (−0.226), and Seychelles (−0.243) also exhibit declines, though less significant, while Cambodia (−0.04), Thailand (−0.065), Viet Nam (−0.087), and Timor-Leste (−0.086) show smaller reductions. The overall trend for SEA shows an APC of −0.176, with a significant increase in ICH incidence in the Philippines.

**Figure 6:**
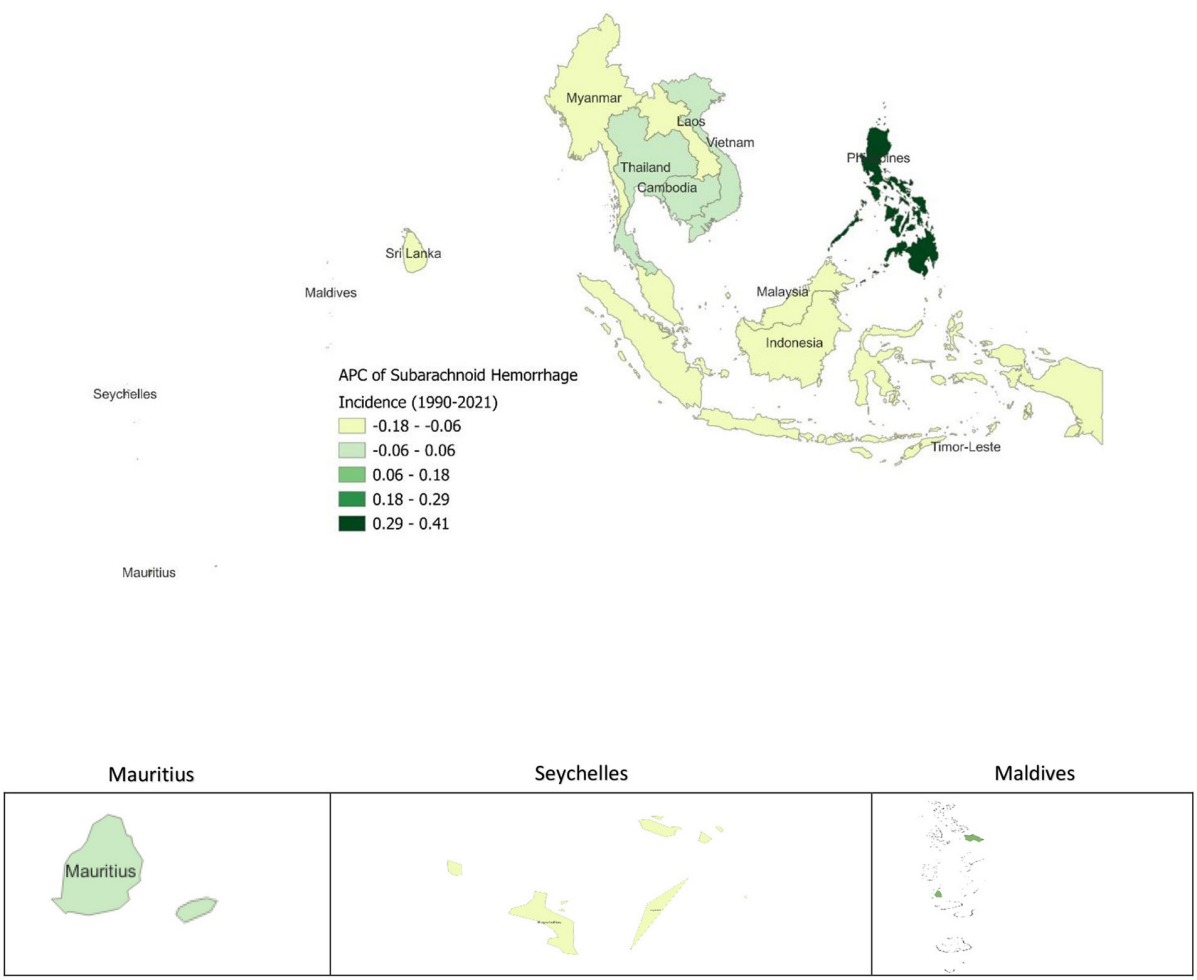
Annual Percentage Change (APC) of Subarachnoid Hemorrhage Incidence from 1990 to 2021.

The analysis of APC in SAH incidence from 1990 to 2021 shows varying trends across SEA. The Philippines has the highest increase in SAH incidence (APC 0.41), while Thailand (0.039) and Cambodia (0.019) also report modest increases (Figure 6). In contrast, many countries show declining trends, with Indonesia (−0.176), Sri Lanka (−0.169), Malaysia (−0.154), and Myanmar (−0.12) recording the largest reductions. Smaller declines are observed in Lao PDR (−0.061), Seychelles (−0.119), Timor-Leste (−0.1), and Vietnam (−0.019). SEA as a whole shows a slight decrease in SAH incidence, with an overall APC of −0.064.

**Figure 7:**
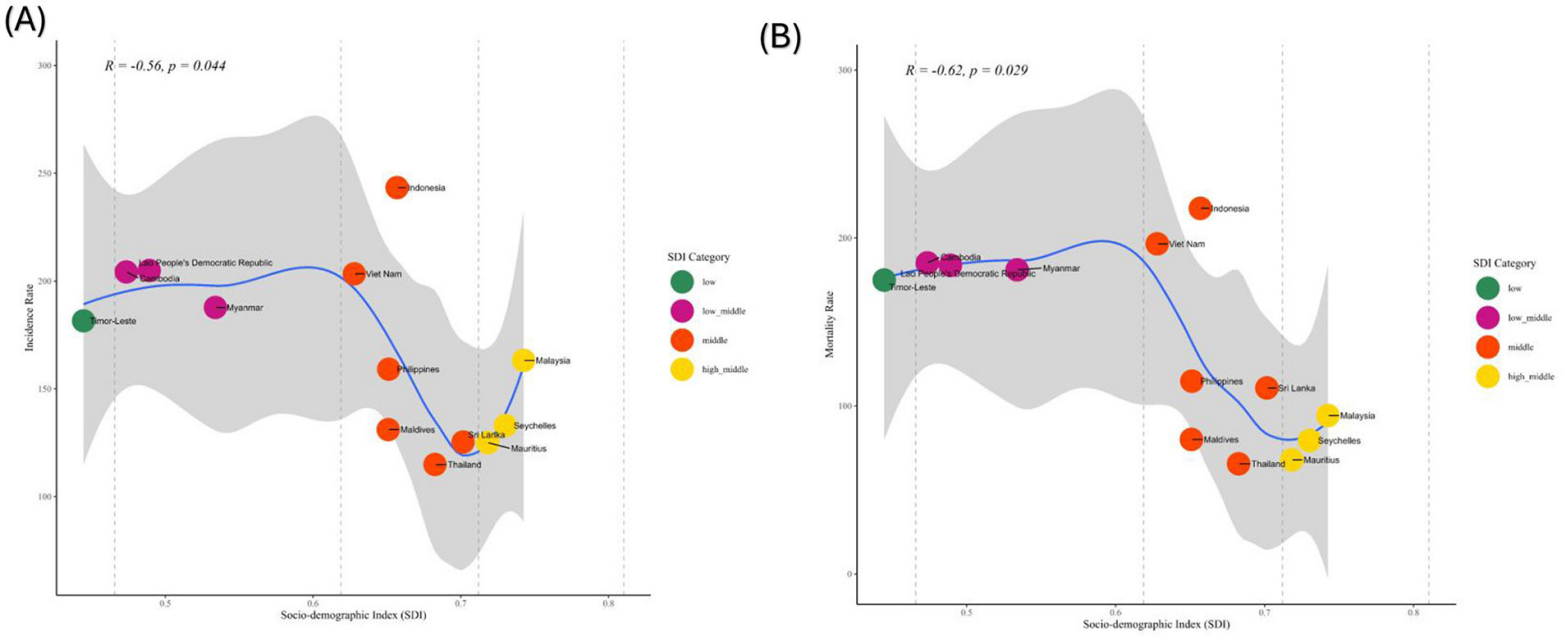
Correlation between the SDI and the age-standardized rate in 2021 in Southeast Asian (SEA) countries. **(A)** Age-standardized incidence rate (ASIR). **(B)** Age-standardized Mortality rate (ASMR); SDI, Socio-demographic Index.

A statistically significant negative correlation was observed between the SDI and the incidence rate of the studied condition (*R* = −0.56, *p* = 0.044). Countries in the low SDI category, such as Timor-Leste, exhibited the highest incidence rates, whereas countries in the high-middle SDI category, including Seychelles and Mauritius, demonstrated the lowest incidence rates (Figure 7A). Variability was observed within individual SDI categories, with some countries displaying incidence rates that deviated from the expected trend based on their SDI classification. A significant inverse correlation was observed between the Socio-demographic Index (SDI) and stroke mortality rates in adolescents and young adults (*R*= −0.62, *p* = 0.029) (Figure 7B). Countries in the low SDI category, such as Timor-Leste, exhibited the highest mortality rates, while countries in the high-middle SDI category, including Seychelles and Mauritius, showed the lowest mortality rates.

## Discussion

The analysis of stroke trends in SEA from 1990 to 2021 reveals distinct patterns across ischemic stroke, ICH, and SAH in younger populations, specifically those aged 15–39. While the total number of cases increased for all three stroke subtypes, the incidence rates, DALYs, and mortality rates show significant improvements, suggesting progress in stroke management and prevention. Across the three stroke subtypes, there was a notable increase in the number of cases between 1990 and 2021. Ischemic stroke cases rose by 34.3%, while ICH and SAH saw increases of 16 and 32.6%, respectively. Despite these rises, both ICH and SAH experienced declines in incidence rates, with ICH decreasing by 17% and SAH showing a 0.2% annual reduction in incidence. These findings are consistent with global trends as noted by Lv et al. (2024), where the ASIR of SAH have decreased over time, despite the overall increase in absolute case numbers in regions with low to middle sociodemographic index (SDI) levels. This reduction may be attributed to targeted health interventions, despite the overall increase in absolute case numbers (Lv et al., 2024). In South-East Asia, stroke represents a critical public health issue, with the region bearing a disproportionately high burden of global stroke-related mortality (Mascarenhas et al., 2024). Moreover, the impact of stroke on adolescents and young adults (15–39 years) remains understudied, despite the growing recognition of its significance within this demographic. The complex interplay of demographic changes, including an aging population, and the rising prevalence of non-communicable diseases like hypertension, diabetes, and cardiovascular conditions has significantly escalated the stroke burden over recent decades. Additionally, widespread lifestyle factors such as tobacco and alcohol use, physical inactivity, and environmental challenges like air pollution and extreme weather exacerbate this issue (Pandian et al., 2023; Collaborators, 2021; Li et al., 2024). Distinctive patterns in the region, including higher rates of hemorrhagic strokes, cerebral venous thromboses, and intracranial atherosclerosis compared to high-income countries (Feigin et al., 2021), highlight the pressing need for targeted, context-specific interventions to address these evolving health challenges effectively.

The DALYs rate, an important indicator of long-term health outcomes, decreased across all three subtypes. ICH exhibited the largest reduction in DALYs (AAPC −0.7158%), followed by SAH (AAPC −0.7475%), while ischemic stroke saw a smaller reduction (AAPC −0.1536%). This suggests that healthcare improvements in SEA, notably in acute care and rehabilitation, have been more effective in reducing the disability burden of hemorrhagic strokes compared to ischemic stroke (Sebastian et al., 2023). Despite an overall increase in the total number of deaths from ischemic stroke, ICH, and SAH, mortality rates have shown significant declines across these subtypes, indicating substantial progress in stroke care for younger populations. In line with findings from Lv et al. (2024), which emphasize that addressing key risk factors such as high systolic blood pressure has likely contributed to reductions in mortality rates for hemorrhagic strokes, our study similarly found that the reduction in mortality was more pronounced for hemorrhagic strokes. Both ICH and SAH experienced sharper declines compared to ischemic stroke, suggesting that targeted interventions for risk factors specific to hemorrhagic stroke may be particularly effective in lowering mortality rates (Lv et al., 2024). In terms of gender, females consistently showed better outcomes across all stroke subtypes. Females saw nearly a 30% decline in DALYs for both ICH and SAH, compared to about 14% for ischemic stroke. This contrasts with males, who experienced a smaller reduction in DALYs and, in some cases, even an increase in ischemic stroke burden. This contrasts with findings from other studies, which underscore that women experience more severe strokes and worse outcomes, even when receiving timely treatment. These differences are influenced by factors such as age, underlying risk factors, and geographical variations (Nahas et al., 2024).

Populations aged 15–39 showed significant reductions in incidence, DALYs, and mortality across all stroke subtypes. DALYs for ICH decreased by over 40%, and similar improvements were observed for SAH and ischemic stroke. These reductions suggest that younger populations have benefited from earlier detection, better management of risk factors, and improved access to healthcare services, particularly in the management of hemorrhagic strokes (Selamat et al., 2022; Turana et al., 2021). Among the age groups within the 15–39 bracket, those aged 30–34 and 35–39 experienced smaller reductions in incidence and DALYs compared to younger age groups. While these cohorts still saw positive trends, the smaller improvements suggest that stroke risk may begin to rise as individuals approach their 40 s, even within the younger demographic. This underscores the importance of maintaining targeted prevention strategies and addressing modifiable risk factors such as smoking, physical inactivity, and hypertension within this age range to continue the positive trend (Zhang et al., 2023; Agianto, 2023). When comparing ischemic stroke, ICH, and SAH, it is evident that while all subtypes saw an increase in total cases, the population-level risk reflected in declining incidence rates for ICH and SAH has generally improved. Hemorrhagic strokes, which are typically more severe, showed the most significant gains in terms of reduced DALYs and mortality, suggesting that healthcare systems have been particularly effective in managing these types of stroke. In contrast, ischemic stroke remains a growing concern, especially among males, who experienced an increase in the disease burden despite the overall improvement in stroke outcomes. This points to a need for more focused interventions to address risk factors like hypertension and smoking, which are prevalent in ischemic stroke (Ng et al., 2019). The substantial improvements among younger populations, especially those aged 15–29, demonstrate the positive impact of public health measures, stroke awareness programs, and better access to acute care in SEA (Chin et al., 2018; Pandian et al., 2023). However, the relatively smaller improvements among the 30–39 age group suggest that more targeted prevention strategies are needed as individuals approach their 40 s, even within this younger population.

The study reveals a significant inverse relationship between the SDI and stroke incidence and mortality rates among adolescents and young adults in Southeast Asia, underscoring the role of socio-economic development in health outcomes. Lower stroke incidence and mortality rates in higher SDI countries, such as Seychelles and Mauritius, can be attributed to better access to acute stroke care, Improved management of modifiable risk factors, and effective prevention strategies (Zhang et al., 2023). Conversely, low SDI countries, face higher rates due to significant obstacles hinder effective stroke care. Limited primary healthcare access restricts early screening and management of stroke risk factors, while a lack of specialized stroke centers and acute care facilities contributes to elevated mortality rates. Furthermore, inadequate public health initiatives and poor awareness of stroke symptoms delay timely interventions, leading to worse health outcomes (Rahbar et al., 2022; Silva et al., 2024). Variability within SDI categories, such as unexpectedly high stroke incidence in Indonesia (a middle-SDI country), highlights the influence of additional factors, including disparities in regional healthcare delivery, behavioral or lifestyle risk factors, and uneven health policy implementation (Adisasmito et al., 2020). Similarly, some high-SDI countries with higher-than-expected mortality may struggle with resource distribution or delays in diagnosis and treatment. These findings emphasize the necessity for tailored public health interventions that address specific country-level challenges while improving socio-demographic conditions. Future research should explore the contextual and systemic disparities influencing these trends to develop targeted, region-specific strategies for reducing the stroke burden in this vulnerable population. The study's main strength is its extensive coverage over a long period (1990–2021) and its unique focus on adolescents and young adults, which provides valuable insights into stroke trends in an often overlooked demographic. Nevertheless, a key limitation is the lack of in-depth analysis of specific risk factors for different stroke subtypes, which limits its ability to offer targeted recommendations.

## Conclusion

The increase in stroke cases across all subtypes in SEA reflects demographic changes and better diagnostic capabilities. Despite this, significant progress in stroke management, particularly for hemorrhagic strokes like ICH and SAH, has led to declines in incidence, DALYs, and mortality, with younger populations benefiting the most. However, ischemic stroke remains a major challenge, especially among males aged 30–39, indicating the need for more targeted interventions. The findings highlight a negative association between SDI and stroke incidence and mortality, with lower SDI countries facing higher burdens. Variability within SDI categories emphasizes the need for context-specific interventions to address disparities and improve outcomes. The success in managing ICH and SAH provides a model for improving ischemic stroke care, with a focus on early intervention and risk factor management, ensuring sustained progress in reducing stroke burdens across the region.

## Data Availability

Publicly available datasets were analyzed in this study. This data can be found here: https://vizhub.healthdata.org/gbd-results/.

## Footnotes

^1^Region AWitS-EA. World Health Organization.
